# Supplementary material for: Study on the effect of a triple cancer treatment of propolis, thermal cycling-hyperthermia, and low-intensity ultrasound on PANC-1 cells
Source: Aging (Albany NY). 2023 Jul 27;15(15):7496–512. doi: 10.18632/aging.204916 (PMC10457055; doi:10.18632/aging.204916)
Supplement: Supplementary Figure 1 [file aging-15-204916-s001.pdf]

SUPPLEMENTARY FIGURE

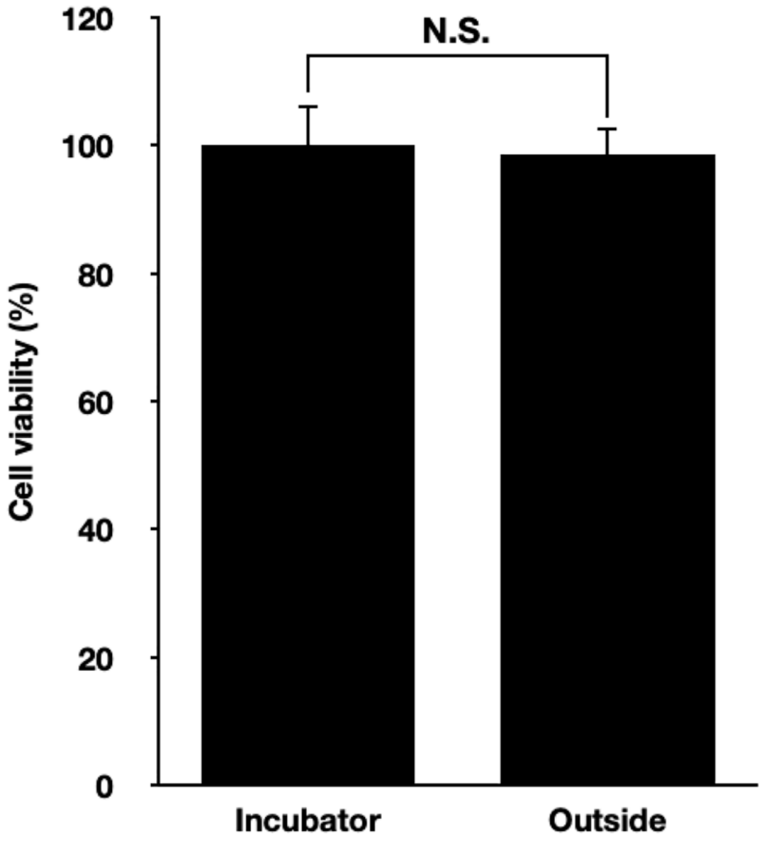

Supplementary Figure 1. Comparison of viabilities of untreated control PANC-1 cells inside or outside the incubator during TC-HT treatment (~45 min). MTT assay was conducted to determine the viabilities of PANC-1 cells after 24 h incubation. Data were presented as the mean ± standard deviation (*n* = 4). N.S. denotes a statistically non-significant difference within the indicated group.
